# Development of the lateral ventricular choroid plexus in a marsupial, *Monodelphis domestica*

**DOI:** 10.1186/1743-8454-7-16

**Published:** 2010-10-05

**Authors:** Shane A Liddelow, Katarzyna M Dziegielewska, John L VandeBerg, Norman R Saunders

**Affiliations:** 1 Department of Pharmacology, the University of Melbourne, Melbourne, 3039, Australia; 2 Department of Genetics and Southwest National Primate Research Centre, Southwest Foundation for Biomedical Research, San Antonio, 78245-0549, USA

## Abstract

**Background:**

Choroid plexus epithelial cells are the site of blood/cerebrospinal fluid (CSF) barrier and regulate molecular transfer between the two compartments. Their mitotic activity in the adult is low. During development, the pattern of growth and timing of acquisition of functional properties of plexus epithelium are not known.

**Methods:**

Numbers and size of choroid plexus epithelial cells and their nuclei were counted and measured in the lateral ventricular plexus from the first day of its appearance until adulthood. Newborn *Monodelphis *pups were injected with 5-bromo-2-deoxyuridine (BrdU) at postnatal day 3 (P3), P4 and P5. Additional animals were injected at P63, P64 and P65. BrdU-immunopositive nuclei were counted and their position mapped in the plexus structure at different ages after injections. Double-labelling immunocytochemistry with antibodies to plasma protein identified post-mitotic cells involved in protein transfer.

**Results:**

Numbers of choroid plexus epithelial cells increased 10-fold between the time of birth and adulthood. In newborn pups each consecutive injection of BrdU labelled 20-40 of epithelial cells counted. After 3 injections, numbers of BrdU positive cells remained constant for at least 2 months. BrdU injections at an older age (P63, P64, P65) resulted in a smaller number of labelled plexus cells. Numbers of plexus cells immunopositive for both BrdU and plasma protein increased with age indicating that protein transferring properties are acquired post mitotically. Labelled nuclei were only detected on the dorsal arm of the plexus as it grows from the neuroependyma, moving along the structure in a 'conveyor belt' like fashion.

**Conclusions:**

The present study established that lateral ventricular choroid plexus epithelial cells are born on the dorsal side of the structure only. Cells born in the first few days after choroid plexus differentiation from the neuroependyma remain present even two months later. Protein-transferring properties are acquired post-mitotically and relatively early in plexus development.

## Background

The choroid plexuses, found in the lateral, third and fourth ventricles of the brain are epithelial tissue masses that are highly vascularised with fenestrated blood vessels. These structures constitute a transfer interface between blood and the cerebrospinal fluid (CSF) which circulates in the ventricular system, subarachnoid spaces and spinal canal. In addition, the choroid plexuses are the main site of CSF production [1,2] and in turn are able to control the homeostasis of its composition by regulating the movement of essential ions and molecules into, and metabolites out of the CSF [3].

The general development of the choroid plexuses has been described before [4-8]. The choroid plexuses in the lateral ventricles themselves do not have a proliferative zone; however, the origin of this organ seems to be the neuroependyma of the ventricular wall at the base of the plexus. From this area the migration of pre-plexus cells can be traced. Once entering the plexus, cells undergo maturation through four distinct stages; described in many different species with the distinct difference in marsupials that glycogen is absent [9].

One of the main functions of the choroid plexus is to regulate the transfer of molecules across blood/CSF interface. For lipid insoluble substances, such as proteins, this transfer has been shown to be across choroid plexus epithelial cells both during development and in the adult [10-16]. However, not all choroid plexus cells seem to be involved in this process, the proportion ranges between less than 5% in the adult to about 15% during early stages of brain development in opossum [11] and rat [12], to over 40% in sheep [13] and humans [14-16]. So far, there is no information available as to when during development plexus cells acquire protein-transferring properties.

In the adult, the proliferation of choroid plexus epithelium has been shown to occur at a very low rate (less than 0.1% of total plexus cells per day [17,18]). Information about the rate and pattern of growth during choroid plexus development is scarce. This study was undertaken in order to investigate the formation of lateral ventricular choroid plexus from the stage when it first becomes clearly differentiated from the neuroependyma. The animal model used was a marsupial, *Monodelphis domestica*, as in this species all of lateral ventricular plexus development occurs postnatally [9,10]. As a result, injections can be made into pups with minimal physiological disturbances of both mother and young.

## Methods

Adult and young postnatal pups of *Monodelphis domestica *(South American grey short-tailed opossum) were used in this study. Animals were obtained from a colony at the Southwest Foundation for Biomedical Research (SFBR) in San Antonio, and conducted according to the PHS Policy on the Humane Care and Use of Laboratory Animals with the approval of the SFBR IACUC. Fixed material was transported to the University of Melbourne where all tissue processing, immunohistochemistry and data analysis were completed.

### Cell Proliferation Study

The lateral ventricular choroid plexus first differentiates at the time of birth in *Monodelphis *[9,10], thus to establish birth date and rate of generation of epithelial cells, pups at postnatal day 3 (P3), P4 and P5 were injected with the thymidine analogue 5-bromo-2-deoxyuridine (BrdU; [19]). Pups received three i.p. injections of BrdU (Sigma, St Louis, MO, USA) 50 mg/kg body weight) at 24 h intervals while still attached to the mother [20]. Injections (approximately 2 μl) were made using a fine glass microcapillary (outer diameter 30-50 μm) containing BrdU dissolved in a sterile 0.9% w/v sterile sodium chloride solution. This injection regime was chosen taking into account the slow growth of cells in the choroid plexus to ensure maximum labelling of epithelial cell nuclei. After the final injection (at P5), animals were left for 2 h, or until they reached P10, P15, P20, P30, P45 or P65. In an additional set of experiments, animals at P63 were injected i.p. with BrdU (50 mg/g body weight) on three consecutive days (ie. at P63, P64 and P65). After the final injection, animals were left for either 2 h or until age P110. At the end of the experiment, animals were terminally anaesthetised with inhaled isoflurane and brains were dissected out and processed for histology, *n *= 6 animals at each age.

### Brain Histology

Dissected brains were immersed in Bouin's fixative for 24 h and washed in 70% ethanol until clean. Tissue was embedded in paraffin wax and 5 μm-thick coronal sections cut through the entire brain and placed on silanized glass slides. Each slide contained between three and seven sections. Every tenth slide was stained with haematoxylin and eosin for routine histology

### Visualisation of incorporated BrdU

Tissue sections were de-waxed by heating to 60°C and placing in histolene baths followed by rehydration through graded ethanol of decreasing concentration and rinsed briefly in phosphate buffered saline (PBS) [11]. To expose nuclear antigen sections were placed in 0.1% sodium citrate buffer and heated in a 1200 W microwave oven for 3 × 3 min (allowing the solution to cool to room temperature between each heating). To neutralise this acidic environment, sections were immersed in PBS + Tween20 for 5 min. Sections were blocked in protein and peroxidase blocking solutions (DAKO, Glostrup, Denmark) for 2 h each at room temperature before being incubated in a mouse monoclonal antibody against BrdU (DAKO; 1:200) for 48 h at 4°C. Following 3 × 5 min washes in PBS + Tween20, sections were incubated for 2 h at room temperature with rabbit anti-mouse immunoglobulin (DAKO, 1:200). After 3 × 5 min washes in PBS + Tween20, sections were incubated with mouse PAP (DAKO, 1:200) for 2 h at room temperature. Following 3 × 5 min washes in PBS + Tween20 and an additional 10 min wash in Tris buffer (0.05 M, pH 7.6) sections were processed with diaminobenzidine (DAB Kit, DAKO) for 5 min. The reaction was halted with a 10 min wash in running distilled water, before sections were dehydrated through graded alcohols and three successive 5 min rinses in histolene, and mounted with Ultramount #4 Mounting Medium (Fronine, Riverstone, NSW, Australia). Control sections were obtained by staining sections from animals that did not receive an injection of BrdU. These control sections always appeared blank.

### Double labelling for BrdU and endogenous plasma protein

Following de-waxing and rehydration (see above), sections were incubated with peroxidase and protein blockers (DAKO) for 2 h in a moist chamber at room temperature. After washing (3 × 5 min) in PBS containing 0.2% Tween20, sections were incubated in a mouse monoclonal antibody against BrdU (DAKO; 1:200) for 48 h at 4°C. Following 3 × 5 min washes in PBS + Tween20, sections were incubated for 2 h at room temperature with rabbit anti-mouse immunoglobulins (DAKO, 1:200). After 3 × 5 min washes in PBS + Tween20, sections were incubated with mouse PAP (DAKO, 1:200) for 2 h at room temperature. Following 3 × 5 min washes in PBS + Tween20 and an additional 10 min wash in Tris buffer (0.05 M, pH 7.6) sections were processed with DAB (DAB Kit, DAKO) for 5 min as above. Sections were returned to PBS briefly (5 min) before incubation with a *Monodelphis*-specific antibody to total plasma protein [21] with added human-specific antibodies to AFP (DAKO) overnight at 4°C (these human-specific antibodies are known to cross-react with endogenous *Monodelphis *AFP, antibodies to AFP, a fetal-specific protein, were added as it is known that antibodies to *Monodelphis *plasma do not contain anti-AFP [11]). Following 3 × 5 min washes in PBS + Tween20, sections were incubated for 24 h at 4°C with a secondary antibody conjugated to Fluorescein (swine anti-rabbit IgG; 1:50, DAKO). After 2 × 5 min washes in PBS + Tween20, a 10 min wash in PBS (no Tween20) and a 10 min wash in Tris buffer, sections were mounted with Fluorescent Mounting Medium (DAKO). Sections were covered and allowed to dry for 20 min before viewing with a fluorescent microscope with appropriate filters attached. Sections were stored, covered, at 4°C. Antibody and reagent dilutions were made in PBS with 2% fish gelatin (Sigma).

### Cell counting

Data in this paper rely on obtaining relatively accurate counts of choroid plexus epithelial cells. A two-dimensional method used was based on choosing pairs of sections no less than 20 μm apart every 200 μm throughout the entire plexus and has been described in detail before [11,12]. This technique provides counts of approximately 10% of the total number of choroid plexus cells at any one age. For simplicity, the term 'total plexus cells' will be used to refer to the 10% of cells actually counted.

### Size of plexus cells and their nuclei

To determine possible developmental changes in size and shape of the choroid plexus epithelial cells and their nuclei, random haematoxylin and eosin stained sections were chosen from 5 separate brains at each age. Sections were viewed and photographed using an Olympus DP70 camera attached to an Olympus BX50 light microscope (10× eyepiece and 40× (0.65NA) objective lens). From each slide, 10 epithelial cells were chosen at random. The cross-sectional area of nuclei, and height, width and cross-sectional area of the choroid plexus epithelial cells in each image was measured using the UTHSCSA Image Tool Software (version 3.00, The University of Texas Health Science Centre in San Antonio). This area measurement was used to determine the volume of each of the structures.

For estimates of nuclear volume the formula of Weibel & Gomez [22] was used:

$$Nuclear\;volume\;(\mu m^{3})=\sqrt{\frac{6}{\pi }}\times {\bar{A}}^{3/2}$$

Where Ā - nuclear profile (μm^2^). This formula assumes that all nuclei are spherical and do not vary greatly within tissue from a single animal. Furthermore, in order to compare the nuclei between different ages it was necessary to ascertain that the shape did not change during development. This was done by measuring the nuclear axial ratio which remained constant - at around 1.00 at all ages. Tissue is known to undergo some volume reduction after Bouin's fixation [23,24], thus the calculated values are probably underestimates of the actual *in vivo *volumes.

### Statistical analysis

Two tailed Student's t test, or one-way analysis of variance (ANOVA), with *post hoc *tests as appropriate were completed with the aid of GraphPad Instat software (version 3.06; GraphPad Software, La Jolla, CA, USA). A *p *value < 0.05 was considered a significant result. All data are expressed as mean ± SEM.

### Photography

All digitized photographs were taken with an Olympus DP70 camera attached to an Olympus BX50 light microscope and processed in Adobe Photoshop CS3^® ^(Adobe ^® ^Systems Incorporated, USA).

The brightness and curve functions were used to obtain images with background close to black or white and to enhance contrast. For co-localisation studies of double-labelled sections, BrdU stained sections that were visualised with DAB were converted to greyscale, inverted and then false coloured red. The overlay function was also used to create final bi-fluorescent images. No other manipulation of the images was conducted.

## Results

### Size and shape of choroid plexus epithelial cells during development

The height of the choroid plexus epithelial cells decreased from approximately 14 μm in P1 to P5 pups, to around 10 μm at P45 and older animals (*p *< 0.05, Table 1). This decrease in height was accompanied by an increase in the width of the epithelial cells, from approximately 7 μm (up to P9) to over 9 μm from P45 onwards (*p *< 0.05). These two cellular shape changes have been described in the literature for other species (eg. mouse [6,7]) with cells developing from columnar early in development to a more cuboidal form in adults. There was also a 50% increase in the volume of the choroid plexus epithelial cells during development, from 656.3 ± 40.7 μm^3 ^at P1, to over 1000 μm^3 ^at P65 (*p *< 0.05, Table 1).

**Table 1:** Size and volume of lateral ventricular choroid plexus epithelial cells throughout *Monodelphis *development

Age	*n*	Height (μm)	Width (μm)	Volume (μm^3^)
**P1**	5	13.7 ± 0.4	6.8 ± 0.2	656.3 ± 40.7
**P5**	5	14.8 ± 0.5	7.8 ± 0.2	942.3 ± 63.9
**P9**	5	14.8 ± 0.3	7.8 ± 0.3	953.4 ± 93.0
**P45**	5	10.6 ± 0.2	9.00 ± 0.2	880.4 ± 44.8
**P65**	5	11.3 ± 0.3	9.4 ± 0.2	1005.4 ± 47.1
**P110**	5	9.9 ± 0.3	9.2 ± 0.3	890.9 ± 65.0
*p*-value*		< 0.05	< 0.05	< 0.05

Data are expressed as mean ± SEM. *n *refers to the number of brains from which choroidd plexus epithelial cells were measured at each age (10 cells from 5 separate brains were measured). *comparing youngest and oldest ages.

### Nuclei of choroid plexus epithelial cells during development

There was no significant change in the radius of nuclei at any age, which varied between 2.5 and 3.0 μm. There were some fluctuations in the volume of the nuclei throughout development, ranging from approximately 90 μm^3 ^(at P1) to just over 120 μm^3 ^(at P9). There was however no statistical difference in the volume of nuclei in plexus cells between the youngest ages and adult (*p *> 0.05). The constancy of the nuclear size was important in the design of a cell counting protocol. As sections used for cell counting were 5 μm thick, no two sections were any closer than 20 μm, and a cellular and nuclear profile had to be visible, there was no likelihood of counting any cell more than once.

### Total number of choroid plexus epithelial cells

In the first days of life in *Monodelphis *the numbers of choroid plexus cells counted increased from about 1500 at P3, to over 2000 at P4 and to over 2500 at P5 (*p *< 0.05, Table 2). The rate of growth was subsequently slower from this age onwards, and by P10 the number counted was just over 3000. By P30 plexus cells counted increased to over 7500 cells and to over 10000 by P45 and P65 (*p *< 0.05). There was a further increase (although not statistically significant) in total plexus cells to 16450 ± 1488 by adulthood (P110).

**Table 2:** Numbers of choroid plexus epithelial cells during development, including numbers positive for plasma protein and BrdU

Age	*n*	Total number of CPECs counted	PP +ve CPECs	BrdU +ve CPECs	PP/BrdU double-labelled CPECs (*n *= 4)
P3	6	1855 ± 260	131 ± 36	44 ± 3	0
P4	6	2060 ± 125	175 ± 12	59 ± 7	2 ± 1
P5	6	2675 ± 205	216 ± 33	76 ± 12	6 ± 2
P10	6	3254 ± 106	230 ± 45	65 ± 17	11 ± 2
P30	6	7790 ± 928	695 ± 113	71 ± 10	-
P45	6	11104 ± 874	512 ± 84	80 ± 16	24 ± 4
P65	6	12175 ± 954	502 ± 54	63 ± 7	39 ± 2
p value *		< 0.05	< 0.05	< 0.05	< 0.05

Data are mean ± SEM, rounded up to the nearest whole cell, *n *values refer to the number of animals in each experiment. Cell count data represent the number of cells counted, not the total number of cells in the plexus. At each age, approximately 10% of the entire plexus was counted. For double-labelling experiments, four separate brains were used (see parenthesis). * comparing youngest and oldest ages in each instance. Abbreviations: BrdU, 5-bromo-2-deoxyuridine; CPECs, choroid plexus epithelial cells; P, postnatal days; PP, plasma protein.

### BrdU labelling of choroid plexus epithelial cells

This study aimed to investigate the development of the lateral ventricular choroid plexus focusing on timing of mitosis and migration of epithelial cells after division from the neuroependymal wall of the ventricles. This was achieved by injecting BrdU i.p. over three days (P3, P4 and P5) and processing brains for histology at different times after, from 2 h to 2 months (see Methods)

**Figure 1:**
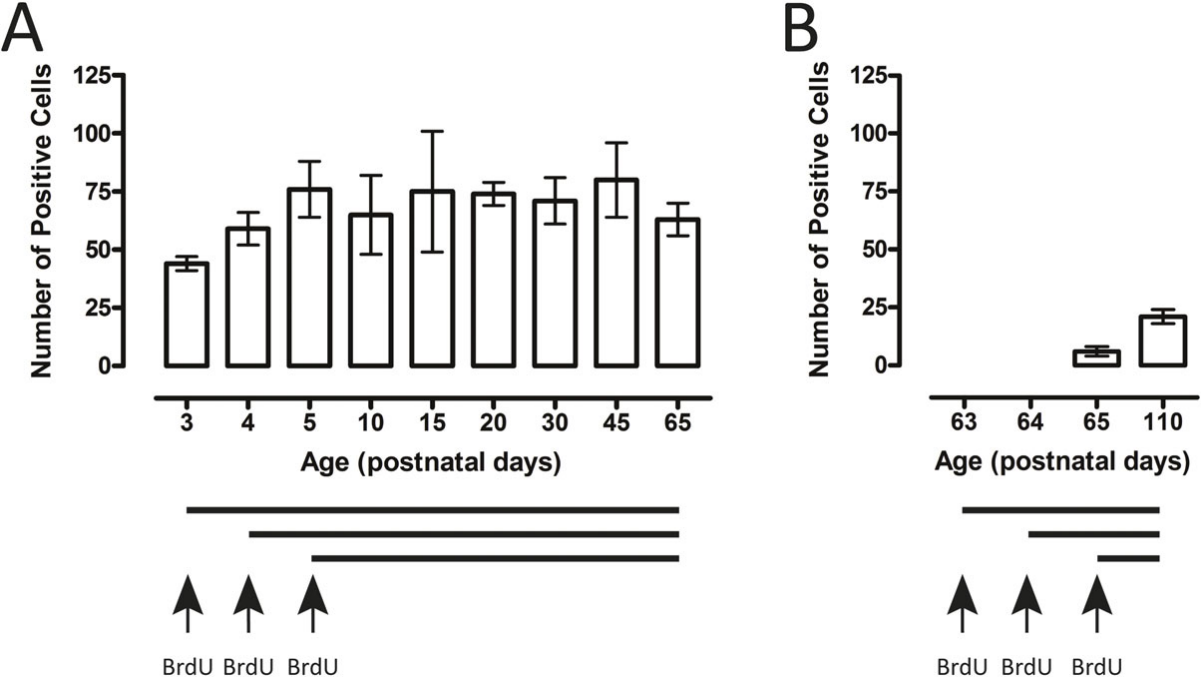
**BrdU labelled cells in the lateral ventricular choroid plexus during development of *Monodelphis***. **A**. Numbers of BrdU positive nuclei of the choroid plexus epithelial cells increased with each consecutive injection. After 3 injections of BrdU into young animals (P3, P4, P5) the number of labelled nuclei remained constant, suggesting a lack of significant turnover of the plexus during this time. **B**. Following injections into older animals (P63, P64, P65) fewer labelled cells were present, suggesting that there is little further growth of the plexus from P65 onwards. *n *= 6 at each age.

After the first injection at P3, about 40 labelled cells were counted (Figure 1 and Table 2). The number of choroid plexus nuclei counted positive for incorporated BrdU increased by approximately 20 with each additional injection, up to 60 at P4 and finally to 76 ± 12 cells at P5 - after the third and final injection (Table 2). From this age onwards there was no change in the number of positive nuclei counted, even up to 2 months of age, with the numbers remaining constant in a range from 65 - 80 (Table 2 and Figure 1A).

In order to study the proliferative ability of plexus epithelium at older ages, in a different set of experiments, animals were injected with BrdU at P63, P64 and P65 and left for 2 h or for another two months (until P110). In these experiments the number of nuclei stained positive for incorporated BrdU counted was around 10 - 20 cells both in P65 and P110 animals (Figure 1B). These low counts are indicative of very few new cells being added to the plexus after P65; this is in agreement with the small increase in numbers of total plexus cells seen after that age (Table 2).

### Position of BrdU labelled cells during lateral ventricular choroid plexus development

**Figure 2:**
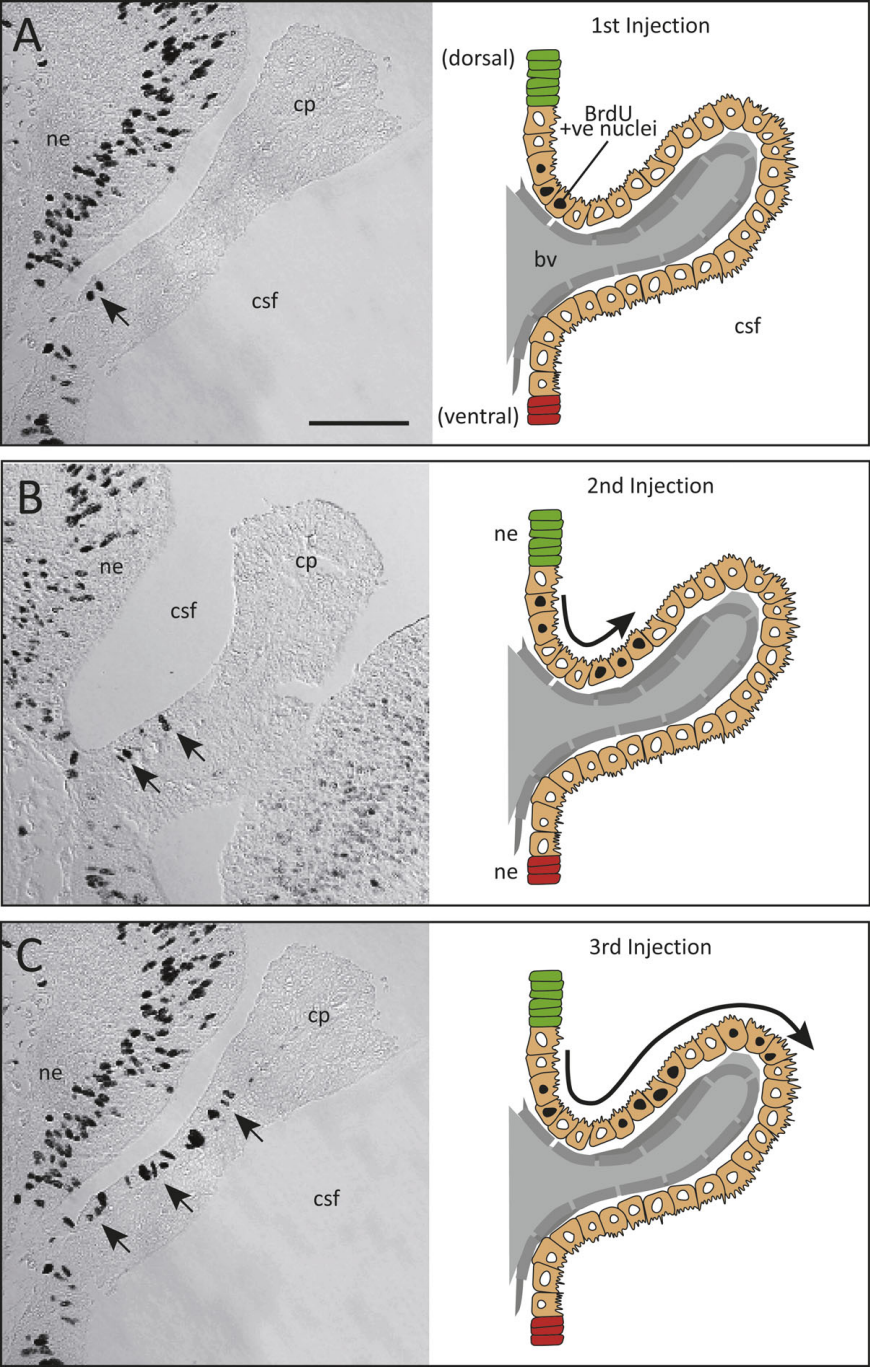
**Progression of BrdU labelled cells along the lateral ventricular choroid plexus during development**. **A**. Coronal section for P5 *Monodelphis *pup after a single injection of BrdU. Positive nuclei were seen in choroid plexus epithelial cells at the root of the plexus (arrow). **B**. After a second injection (24 h later) two distinct labelled populations of nuclei were seen on the same side (dorsal) of the plexus (arrows). **C**. Three consecutive injections of BrdU 24 h apart show nuclei of choroid plexus epithelial cells being 'pushed' along the stalk of the plexus, away from the root, by newly dividing cells of the structure. *Camera lucida *images on the right depict the movement of the nuclei as the plexus increases in size. Scale bar: 50 μm for all. Abbreviations: bv, blood vessel; cp, choroid plexus; csf, cerebrospinal fluid; ne, neuroependyma.

The position of BrdU labelled cells within the plexus structure was monitored following injection. The aim was to investigate how choroid plexus cells migrate once entering the structure and to determine if cell birth occurs at both ends of the choroid plexus root as it originates from the neuroependyma. The results show that after injection of BrdU, labelled nuclei were found near the root of the choroid plexus, on the upper (dorsal) arm only, with no positive cells found on the lower (ventral) surface (Figure 2). Following successive injections, distinct populations of labelled nuclei were seen, indicative of each individual injection of BrdU (arrows in Figure 2). In addition, with increased time after injection the normal growth of the plexus caused epithelial cells with labelled nuclei to be pushed out from the root of the plexus towards the tip (*camera lucida *images in Figure 2). The length of time between the incorporation of BrdU and the distance to where positive nuclei along the plexus stalk were found (an indicator of plexus growth) appears to be linearly correlated - thus with a longer time period, labelled cells were found further along the choroid plexus, away from its origin in the ventricular zone (Figure 2).

In animals injected at an older age (P63, P64 and P65), labelled nuclei were again seen dorsally, at the root of the plexus and never at the tip.

These observations suggest that growth of the lateral ventricular choroid plexus occurs only from its root, and only on the dorsal surface of the structure.

### Co-localization of BrdU and endogenous plasma protein

Many of the proteins found in CSF both during development and in the adult are thought to originate mostly from blood plasma [11,12,25-27] by a transfer mechanism that is intracellular [11,27-30], across only a small proportion of the plexus epithelium [11,12,26]. In order to establish the relationship between protein transferring properties and the time of birth of choroid plexus cells, double labelling immunocytochemistry was used on tissue sections from animals injected with BrdU. These results are included in Table 2 together with total numbers of plexus cells and numbers of plasma protein positive cells.

**Figure 3:**
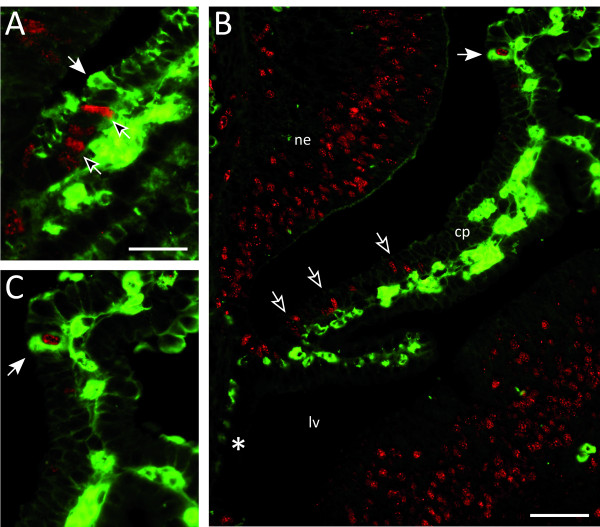
**Fluorescent double labelling of endogenous plasma protein and BrdU**. **A. **P3 *Monodelphis *2 sh after a single injection of BrdU. Nuclei positive for the cell division marker BrdU are visible (unfilled arrows, red nuclei), as well as choroid plexus epithelial cells positive for plasma protein (filled arrow, green cells). Nuclei were seen only to appear from the root on the upper (dorsal) side of the plexus. There were no positive nuclei further along the plexus than protein positive cells. No plexus cells positive for protein were double-labelled for BrdU at this age. **B**. P10 *Monodelphis *injected with BrdU at P3, P4 and P5. BrdU positive nuclei (unfilled arrows, red nuclei) were seen further along the stalk of the plexus. Some cells were positive for plasma protein (green) and double labelled with BrdU (filled arrow). BrdU positive nuclei were only seen on one side of the plexus. No positive nuclei were seen in the root on the contralateral side (asterisk). **C**. Higher power image of double-labelled cell in **B**. Abbreviations: cp, choroid plexus; lv, lateral ventricle; ne, neuroepithelium. Scale bar: 25 μm in A, C; 50 μm in B.

Following one injection of BrdU at P3, nuclei of 44 ± 3 cells with labelled nuclei were counted. At the same age 131 ± 36 plexus cells counted were plasma protein positive, however none were double labelled (Table 2 and Figure 3). In animals injected, at P3 and P4, about 60 BrdU positive nuclei, 175 ± 12 plasma positive cells and only 2 ± 1 double labelled cells were counted. At P5 (after the third BrdU injection) the number of positive nuclei counted was 76 ± 12 and plasma protein positive cells was 216 ± 33. The number of double labelled cells counted was 6 ± 2 at P5 and 11 ± 2 by P10 (Table 2 and Figure 3). From P5 until P65 the numbers of BrdU labelled nuclei counted did not change (60-80), plasma protein positive cells increased from about 250 to 500-600 and double-labelled cells increased to 24 ± 4 at P45 and to 39 ± 2 at P65. The proportion of double labelled cells in relation to BrdU positive cells increased from 2-3% at P3 and P4, to about 10% after the third at P5, to nearly 20% at P10, 30% at P45 and over 50% at P65.

This indicates that the protein transferring properties of plexus epithelial cells are acquired post-mitotically but also that a large proportion of cells born during the early period of plexus growth become protein transferring (Table 2).

## Discussion

The results obtained in the present study demonstrated that there is an increase in the number of choroid plexus epithelial cells, due to mitotic division, during normal development of the marsupial *Monodelphis*. In addition, the results show that these cells are only added to the plexus from one side only (dorsal) and once they become choroid plexus epithelial cells, they do not seem to divide again. However, cells do continue to migrate as a result of newly developing cells being added, causing them to be pushed outwards like a 'conveyor belt' along the stalk of the plexus, further away from the ventricular wall.

The development of the plexus epithelial cells is generally divided into four stages [4,6] and these in *Monodelphis *are:

Stage I: the epithelial cells are pseudostratified with centrally located nuclei. In *Monodelphis *the majority of plexus cells in this stage are found from the day of birth up until about P12.

Stage II: the choroid epithelium is low columnar to cuboidal in shape. The basal connective tissue is beginning to form, and the apical villi are not present. Nuclei are moving towards the apical surface of the cell. These cells are visible from around P13 in *Monodelphis*. Most noticeably, compared to eutherian species, glycogen is not present in marsupial choroid plexus epithelial cells [10].

Stage III: this stage (P30 - P45 in *Monodelphis*) is characterized by cuboidal epithelial cells with nuclei towards the basal surface. It is in this stage that the cilia on the apical surface of the cell appear and there is a great increase in the complexity of the capillary network, with an accompanied increase in the number of villi [31].

Stage IV: in the final stage of plexus epithelial cell development, visible from about P45 onwards in *Monodelphis*, the cuboidal cells become slightly smaller (approximately 10 μm^2 ^in profile), with most nuclei situated centrally to basally within the cytoplasm.

Very few systematic studies have been completed on the histogenesis of the epithelial cells of the choroid plexus. Due to its early appearance and large size, the lateral ventricular choroid plexus has been the most studied. It is widely accepted that it arises from an infolding of the multilayered roof plate of the neural tube, between the paraphyseal arch and the medial wall [4,6,32-36]. It has been shown using chick-quail chimeras [37] that the specific cells destined to become the beginnings of the choroid plexus are detectable up to 3 days before the structure even emerges from the ependymal wall of the ventricles. Indeed, this 'pre plexus' ependyma is induced to envelope connective tissue and develop blood vessels and hence a plexus, upon coming into direct contact with tissue derived from the mesodermal germ layer [38].

This area, which is originally formed by an invagination of the anterior end of the neural tube [39], then divides into three regions: the first (and most lateral) gives rise to the cortical neuroepithelium, the origin of neurons and glia of the cortex; the second (medial or dorsal telencephalic midline region) is split in two, with the cortical hem being a major source of Cajal-Retzius cells of the neocortex; and, the medial region of the dorsal telencephalic midline region gives rise to the choroid plexus epithelium.

The medial-lateral patterning of the dorsal region of the telencephalon is regulated by a large number of transcription and secreted signalling factors. Some examples of such secreted factors include members of the bone morphogenic protein (BMP) family regulate specification of the choroid plexus epithelium by inducing Msx1 and repressing Lhx2/Foxg1 expression [40-47]. The importance of BMPs in the early development of the choroid plexus has been studied intensely. A lack of the constitutively active form of the receptors for BMPs results in a massive expansion of the choroid plexus epithelium at the expense of the cortical neuroepithelium [45]. In contrast, inactivation of BMP receptors results in retarded growth of the choroid plexus, specifically of the epithelial cells [46,47].

The proliferative nature of choroid plexus epithelial cells has been shown to occur even after the original dissemination from the neuroependymal wall and a possible neuronal fate. The choroid plexus epithelial cells from rats at a range of developmental ages from postnatal day 1 to 8 weeks have been shown to have an ability to function as neural progenitor cells, however this ability decreases with age, with the plexus epithelium from P1 animals twice as likely to undergo the change than those from adult (8 week) animals [48]. These authors also present data that illustrate the proliferation of choroid plexus epithelium occurring in adult rats, though at a very low rate (less than 0.1% of total plexus cells). This low level of mitotic activity in adult choroid plexus epithelial cells has been reported elsewhere [17,18] and is in agreement with the lower numbers of labelled cells seen in older animals in the current study (which equate to approximately 0.05% of the total plexus at P65, calculated from Table 2).

### Function of choroid plexus epithelial cells

The epithelial cells of the choroid plexus have many functions. They are the site of the blood/CSF barrier, a protective mechanism that ensures the stability of the CSF milieu [1,49]. This barrier is due to the presence of tight junctions between adjacent epithelial cells, junctions that are tight to molecules as small as lanthanum ions (139 Da; [50]) and tracer molecules such as biotin ethylenediamine (286 Da; [10]). The presence of this functional barrier is an essential prerequisite for the establishment and maintenance of concentration gradients for ions and proteins between the blood (basal side of the cells) and the CSF on the apical side [51] that are set up by transport mechanisms in the plexus epithelial cells. Another important function, especially during development, is the ability of the plexus epithelial cells to transfer plasma proteins from blood to CSF [11,12,28,52]. Although the route of this transfer has been identified as intracellular, the actual mechanism remains unknown [11]. Results in the present study demonstrated that the ability to transfer protein across blood/CSF barrier is a post-mitotic phenomenon and that cells involved in this process are born early in plexus development (Table 2). This is supported by the findings that immediately following only one BrdU injection no cells were double labelled with antibodies to plasma protein but increasing numbers of such cells could be identified progressively with age and by P65 about 50% of cells born during the period P3-5 became plasma protein positive, (Table 2 and Figure 3). This is in contrast to the ontogenic development of choroid plexus cells involved in the transfer of passive, exogenous molecules such as dextran amines [10,11]. In a set of preliminary experiments results showed that the ability to transfer passive markers of various molecular sizes (from 3 k to 70 k) is present in the plexus cells immediately upon their birth (Liddelow SA, unpublished observations). This adds further evidence to the proposition that there is more than one transfer mechanism for macromolecules present in the choroid plexus epithelial cells, at least during development [11].

## Conclusions

The current study describes the growth pattern of the lateral ventricular choroid plexus in *Monodelphis domestica*, reporting that it occurs from only one side (dorsal) of the structure. Cells born during the first few days of plexus formation are still present even two months later indicating a low turnover of this tissue. The rate of growth was slower in older animals. The functional ability to transfer protein from blood plasma into the CSF [11] is acquired post-mitotically and many of the protein-transferring cells are born early in plexus development.

## Competing interests

The authors declare that they have no competing interests.

## Authors' contributions

SAL contributed to the design of the study, performed all experiments, data collection and analysis, and drafting of the manuscript. KMD contributed to the design of the study, data analysis and drafting of the manuscript. JLV contributed to the design of the study and drafting of the manuscript. NRS contributed to the design of the study and drafting of the manuscript. All authors have read and approved the final version of the manuscript.
